# miR-3059-3p Regulates Glioblastoma Multiforme Radiosensitivity Enhancement through the Homologous Recombination Pathway of DNA Repair

**DOI:** 10.1155/2022/7250278

**Published:** 2022-09-21

**Authors:** Yu-Wen Cheng, Chien-Ju Lin, Shih-Hsun Kuo, Ann-Shung Lieu, Chee-Yin Chai, Hung-Pei Tsai, Aij-Lie Kwan

**Affiliations:** ^1^Department of Neurosurgery, Kaohsiung Veterans General Hospital, Kaohsiung, Taiwan; ^2^Graduate Institute of Medicine, College of Medicine, Kaohsiung Medical University, Kaohsiung, Taiwan; ^3^School of Pharmacy, College of Pharmacy, Kaohsiung Medical University, Kaohsiung, Taiwan; ^4^Department of Radiation Oncology, Kaohsiung Medical University Hospital, Kaohsiung, Taiwan; ^5^Division of Neurosurgery, Department of Surgery, Kaohsiung Medical University Hospital, Kaohsiung, Taiwan; ^6^Department of Surgery, School of Medicine, College of Medicine, Kaohsiung Medical University, Kaohsiung, Taiwan; ^7^Department of Pathology, Kaohsiung Medical University Hospital, Kaohsiung, Taiwan; ^8^Department of Pathology, School of Medicine, College of Medicine, Kaohsiung Medical University, Kaohsiung, Taiwan; ^9^Department of Neurosurgery, University of Virginia, Charlottesville, VA, USA

## Abstract

**Background:**

Glioblastoma multiforme (GBM) is one of the most deadly and recalcitrant illnesses of the neurocentral nervous system in humans. MicroRNAs (miRNAs) are a class of noncoding RNAs that play important roles in the regulation of gene expression and biological processes, including radiosensitivity. In this study, we demonstrated the relationship between miR-3059-3p and radiation in GBM.

**Materials and Methods:**

Radioresistant (RR) cells were obtained by exposing GBM8401 cells to 80 Gy radiation in 20 weekly 4 Gy fractions. miR-3059-3p mRNA and DNA replication helicase/nuclease 2 (DNA2) protein expressions were detected using real-time polymerase chain reaction and immunoblotting. Using flow cytometry, colony formation and apoptosis were identified using miR-3059-3p mimic, miR-3059-3p inhibitor, DNA2 siRNA, and DNA2 plasmid. Immunoblotting was used to detect DNA repair proteins.

**Results:**

Low levels of miR-3059-3p and high levels of DNA2 were observed in RR cells. Colony formation and apoptosis assays revealed that miR-3059-3p targeted DNA2 to regulate radioresistance. Immunoblotting revealed that miR-3059-3p regulated the homologous recombination (HR) pathway (Rad51 and Rad52) but not the nonhomologous end joining pathway (ku70 and ku80).

**Conclusion:**

Downregulation of DNA2 via miR-3059-3p enhanced the radiosensitivity of GBM cells through the inhibition of the HR pathway.

## 1. Introduction

Glioblastoma multiforme (GBM) in humans is one of the most deadly and recalcitrant illnesses of the neurocentral nervous system. Approximately 12,120 patients in the United States alone were diagnosed with GBM in 2016 with a 5-year survival rate of 5%, and the peak age-adjusted incidence of GBM is estimated to be 3.2 per 100,000 [1]. The exact etiology of the disease is currently unknown, and only limited well-established research has indicated radiation as the cause [2]. Clinical results of GBM may present some obvious symptoms, including persistent weakness, numbness, loss of vision, or changes in language based on the neurological function. As the tumor size increases, symptoms such as headache, nausea, vomiting, and even loss of consciousness also appear. Magnetic resonance imaging is the standard radiographic imaging modality in the diagnosis and posttreatment management of patients with glioblastoma [3]. Current treatment approaches include surgical resection with radiotherapy (RT) as well as concomitant and maintenance chemotherapy, such as temozolomide [4]. The overall survival is still dismal, and the average survival time is <2 years [5, 6]. Despite advancements in neurosurgery and RT, the development of potent chemotherapeutic drugs, and comprehensive genomic profiling and molecular diagnostics over the last several decades, there has been little improvement in increasing the overall survival rate [7].

Radioresistance (RR) is responsible for the poor therapeutic effect of RT on GBM tumors. GBM cells exhibit increased proliferation and insufficient vascularization, which induces local hypoxia in tumor sites [8, 9]. Moreover, hypoxia is well known to play an important role in RR. Additionally, fractionated RT, epithelial–mesenchymal transition, and cancer stem cells can induce RR [10, 11]. Therefore, inhibiting RR can improve the therapeutic effect of RT on GBM tumors.

MicroRNAs (miRNAs) are noncoding RNAs that play an important role in regulating mRNA expressions. The average length of a miRNA molecule is 22 nucleotides. They are transcribed from DNA sequences into primary miRNAs (pri-miRNAs) and processed into precursor miRNAs (pre-miRNAs), which then mature into miRNAs. miRNAs have been shown to regulate gene expressions following binding to the 3′-untranslated region of target mRNAs to induce mRNA degradation or translational repression [12–14]. Recent studies have shown that miRNAs can regulate RR by targeting mRNAs to mediate many biological processes, including proliferation, cell cycle, aging, apoptosis, and DNA repair [15–19]. Several studies have shown that microRNAs can regulate the therapeutic effect of radiation. For example, miR-409-3p mediated radiosensitivity in non-small-cell lung cancer [20]. miR-31 induced RR by regulating reactive oxygen species in pancreatic ductal adenocarcinoma [21]. In addition, the tumor environment is associated with radioresistance. In colorectal cancer, exosomal miR-590-3p from cancer-associated fibroblasts regulated radioresistance [22]. At present, miR-3059-3p has been shown to regulate stress-induced depression and resilience [23]. However, there are no reports on the relationship between miR-3059-3p and radiation. This study is therefore aimed at investigating the relationship between miR-3059-3p and radiosensitivity and the underlying mechanisms.

## 2. Materials and Methods

### 2.1. Cell Culture

GBM8401cells were obtained from the Bioresource Collection and Research Center and cultured in RPMI medium supplemented with 10% fetal bovine serum under 5% CO_2_ atmosphere at 37°C. The cells were exposed to 80 Gy radiation in 20 weekly 4 Gy fractions to yield RR cells.

### 2.2. Colony Formation

GBM cells were seeded into 6-well plates at a density of 100, 200, 400, 1000, and 10,000 cells per well and exposed to radiation doses of 0, 1, 2, 4, and 8 Gy, respectively. A linear accelerator was used to irradiate cells, which was performed at room temperature. The cells were stained with 0.5% crystal violet after a 10-day incubation. The number of colonies formed was normalized to plating efficiency (PE) and represented as a surviving fraction (SF) relative to the control. The PE and SF were calculated as follows: PE = (number of colonies formed/number of inoculated cells) × 100%; SF = number of colonies formed/(number of seeded cells × [PE/100]).

### 2.3. Transfection

MicroRNA was transfected into GBM8401 cells using DharmaFECT transfection reagents (Dharmacon™, Lafayette, USA). Transfection was performed using 5 *μ*M microRNA mimic/inhibitor or DNA replication helicase/nuclease 2 (DNA2) siRNA/plasmid for 2 days. The following microRNAs were used: miRNA mimic negative control sense UCACAACCUCCUAGAAAGAGUAGA, miRNA mimic negative control antisense UCUACUCUUUCUAGGAGGUUGUGA, miRNA inhibitor negative control antisense UCUACUCUUUCUAGGAGGUUGUGA, miR-3059-3p mimic sense CCUCUAGGGAAGAGAAGGUUGG, miR-3059-3p mimic sense CCAACCUUCUCUUCCCUAGAGG, and miR-3059-3p inhibitor antisense CCAACCUUCUCUUCCCUAGAGG.

### 2.4. MicroRNA Polymerase Chain Reaction (PCR)

MicroRNAs were extracted and purified using the miRNeasy Mini kit (Qiagen, Hilden, Germany). miRNA expression levels were measured via quantitative reverse transcription- (qRT-) PCR using StepOne (Thermo, Waltham, USA). The cycling conditions were as follows: 95°C for 10 min, followed by 40 cycles of amplification at 95°C for 15 s, and 60°C for 60 s. The relative miR-3059-3p expression level was calculated using the 2^−*ΔΔ*Ct^ method. U6 was used as an internal control.

### 2.5. Immunoblotting

The cells were lysed with RIPA buffer, and 50 *μ*g of protein per sample was loaded into the wells of a 10%–12% sodium dodecyl sulfate–polyacrylamide gel electrophoresis gel and electrophoresed at 70 and 110 V for 1 h each. The proteins were transferred to a polyvinylidene fluoride membrane following electrophoresing at 400 mA for 2 h. The membranes were blocked with blocking buffer for 1 h and incubated overnight with the respective primary antibody (*β*-actin (1 : 20,000; Sigma-Aldrich; A5411), Rad51 (1 : 1000; GeneTex; GTX70230), Rad52 (1 : 1000; SANTA CRUZ; sc-365341), Ku70 (1 : 1000; Arigo; ARG57851), Ku80 (1 : 1000; Arigo; ARG57867), and DNA2 (1 : 1000; Merck; HPA057526)) at 4°C, followed by incubation with the corresponding secondary antibody (goat anti-rabbit (1 : 5000; Millipore; AP132P) and goat anti-mouse (1 : 5000; Millipore; AP124P)) for 90 min. An enhanced chemiluminescence solution (Western Lightning; 205-14621) was used for detecting specific bands using the Mini Chemiluminescent Imaging and Analysis System (MINICHEMI; Beijing; China).

### 2.6. Flow Cytometry

A total of 1.5 × 10^5^ GBM8401 cells were seeded into 6-well plates and incubated for 24 h followed by transfection with microRNA mimic, microRNA inhibitor, DNA2 siRNA, or DNA2 plasmid for 48 h and exposed to radiation. Both detached and attached cells were centrifuged at 1500 rpm for 5 min. Cells were washed once with phosphate-buffered saline and analyzed using the Muse® Annexin V and Dead Cell Assay Kit (Millipore, MCH100105, Burlington, USA).

### 2.7. Data Analysis

The SPSS 24.0 (IBM, NY, USA) software was used for statistical analysis. A one-way analysis of variance followed by Tukey's post hoc test was used to analyze the results of colony formation, apoptosis percentage, and western blot. For all analyses, a *P* value of < 0.05 was considered statistically significant.

## 3. Results

### 3.1. miR-3059-3p Expression in RR GBM Cells

**Figure 1:**
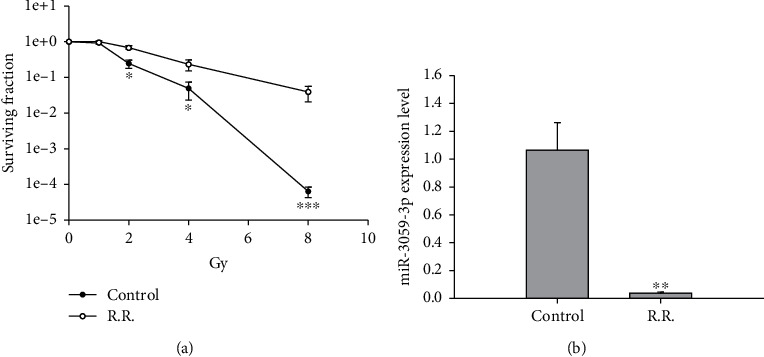
Colony formation and miR-3059-3p expression in RR cells. (a) Comparison of the surviving fraction between the RR and control groups receiving 0, 1, 2, 4, and 8 Gy radiation. (b) Real-time polymerase chain reaction was performed to analyze the expression level of miR-3059-3p, which was compared between the RR and control groups. ^∗^*P* < 0.05, ^∗∗^*P* < 0.01, and ∗∗∗*P* < 0.001 compared with controls. RR: radioresistant.

To confirm the effect of miR-3059-3p on RR cells, we first evaluated SF under 0, 1, 2, 4, and 8 Gy radiation between the control and RR groups in GBM8401 cells. The results revealed that the RR group had a higher SF than the control group at 2 (*P* < 0.05), 4 (*P* < 0.05), and 8 (*P* < 0.001) Gy radiation (Figure 1(a)). miR-3059-3p expression was also detected in both groups, as revealed through qRT–PCR, where the RR group exhibited lower miR-3059-3p expression levels than the control group (*P* < 0.01) (Figure 1(b)). Therefore, miR-3059-3p may play an important role in radiosensitivity.

### 3.2. miR-3059-3p Enhanced Radiosensitivity in GBM8401 Cells after Radiation

**Figure 2:**
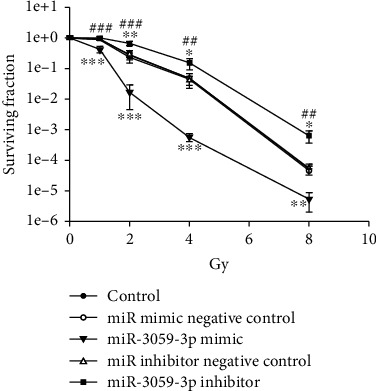
The surviving fraction of irradiated GBM cells with miR-3059-3p mimic or inhibitor. Comparison of the surviving fractions between the control, miR-3059-3p mimic, and miR-3059-3p inhibitor groups using colony formation assays in GBM8401 cells. ^∗∗^*P* < 0.01 and ^∗∗∗^*P* < 0.001 compared with the control group. ^##^*P* < 0.01 and ^###^*P* < 0.001 compared with the miR-3059-3p mimic group. GBM: glioblastoma multiforme.

**Figure 3:**
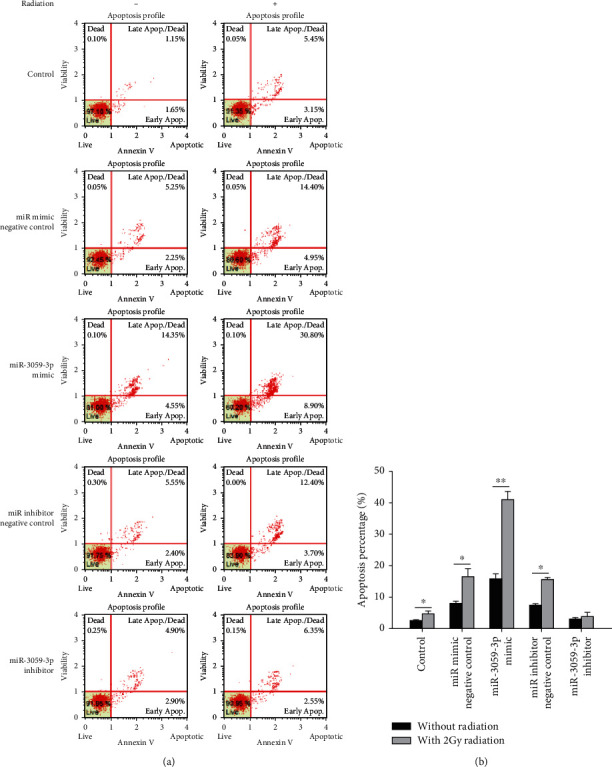
Apoptosis assay for miR-3059-3p with radiation. (a) Apoptosis of cells was determined via flow cytometry. (b) The percentage of apoptotic cells. ^∗^*P* < 0.05 and ^∗∗^*P* < 0.01.

The role of miR-3059-3p in radiosensitivity was further investigated. We evaluated the SF after radiation in miR-3059-3p mimic, miR-3059-3p inhibitor, miR mimic negative control, miR inhibitor negative control, and control groups in GBM8401 cells. The SF did not differ significantly between the control, miR mimic negative control, and miR inhibitor negative control groups. Compared with the other mimics, inhibitors, and controls, the miR-3059-3p inhibitor increased the number of colonies formed while miR-3059-3p mimic decreased it at 1 (*P* < 0.001), 2 (*P* < 0.001), 4 (*P* < 0.001), and 8 (*P* < 0.001) Gy radiation (Figure 2). These findings showed that miR-3059-3p regulated radiosensitivity in GBM cells. To confirm the therapeutic effect of radiation with miR-3059-3p, an apoptosis assay was performed in GBM8401 cells exposed to radiation using flow cytometry. The results indicated that the miR-3059-3p mimic group had a greater percentage of apoptotic cells than the control and miR-3059-3p mimic negative control groups after 2 Gy radiation (Figure 3(a)). The apoptosis percentages of the control, miR mimic negative control, miR-3059-3p mimic, miR inhibitor negative control, and miR-3059-3p inhibitor groups without radiation were 2.57% ± 0.21%, 7.9% ± 0.76%, 15.8% ± 1.59%, 7.3% ± 0.54%, and 3.0% ± 0.49%, respectively. After 2 Gy radiation, these values were 4.6% ± 0.93%, 16.4% ± 2.64%, 40.9% ± 2.71%, 15.5% ± 0.74%, and 3.8 ± 1.42%, respectively. Afterward, the miR-3059-3p mimic group showed significantly higher apoptosis than the other groups with 2 Gy radiation. In the control, miR mimic negative control, and miR inhibitor control groups, 2 Gy radiation led to a twofold increase in the percentage of apoptosis cells. Transfection with miR-3059-3p mimic resulted in a 2.59-fold increase in apoptosis cells after 2 Gy radiation. However, transfection with miR-3059-3p inhibitor only showed a 1.25-fold increase (Figure 3(b)). These data suggest that miR-3059-3p can regulate radiation-induced apoptosis.

### 3.3. Mechanism of miR-3059-3p-Mediated Effects in RR GBM Cells

**Figure 4:**
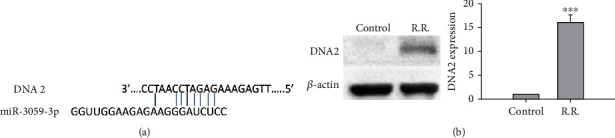
The target of miR-3059-3p. (a) The binding relation between miR-3059-3p and DNA2 was predicted using miRDB, an online database for miRNA target prediction and functional annotations. (b) In immunoblotting, the RR group showed higher DNA2 expression. ^∗∗∗^*P* < 0.001 than the control group.

**Figure 5:**
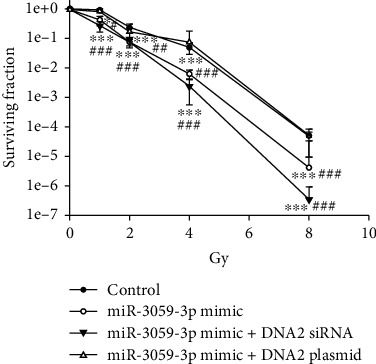
The miR-3059-3p mimic + DNA2 siRNA group showed a significantly low survival percentage. ^∗^*P* < 0.05 and ^∗∗∗^*P* < 0.001 compared with the control group. ^##^*P* < 0.01 and ^###^*P* < 0.001 compared with the miR-3059-3p + DNA2 plasmid group.

**Figure 6:**
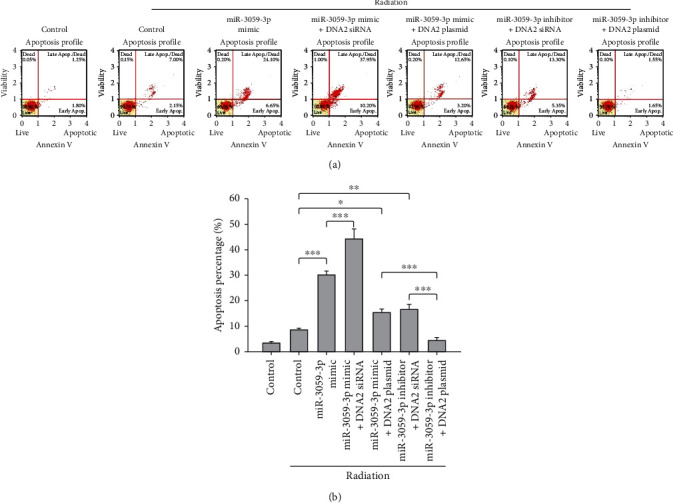
Apoptosis assay for miR-3059-3p and DNA2 with radiation. (a) Apoptosis of cells was determined by flow cytometry. (b) The percentage of apoptotic cells. The miR-3059-3p mimic with DNA2 siRNA group showed a higher apoptosis percentage than the other groups. ^∗^*P* < 0.05, ^∗∗^*P* < 0.01, and ^∗∗∗^*P* < 0.001.

We further predicted the presence of a binding site between DNA2 and miR-3059-3p using miRDB [24], an online database for miRNA target prediction and functional annotations (Figure 4(a)), to clarify the role of DNA2 in RR *in vitro*. Moreover, DNA2 expression patterns in GBM8401 cells were detected using immunoblotting, and a higher DNA2 expression level was found in the RR group than in the control group (*P* < 0.001) (Figure 4(b)). To confirm the mechanism of miR-3059-3p in radiation, we transfected DNA2 siRNA or plasmid including miR-3059-3p mimic into GBM8401 cells and then evaluated the SF after radiation. miR-3059-3p mimic group showed a lower SF than the control group, as described above. The miR-3059-3p + DNA2 siRNA group exhibited the lowest SF, and the miR-3059-3p with DNA2 plasmid rescued the SF, with the level being nearly identical to that of the control group (Figure 5). We analyzed the apoptosis fraction among different combinations of miR-3059-3p and DNA2 after radiation exposure using flow cytometry. We found an increase in the percentage of apoptotic cells after radiation, which confirmed the effect of DNA2 downregulation (Figure 6(a)). The control group had 3.4% ± 0.56% apoptotic cells. After 2 Gy radiation, the percentages of apoptosis cells in the control, miR-3059-3p mimic, miR-3059-3p mimic + DNA2 siRNA, miR-3059-3p + DNA2 plasmid, miR-3059-3p inhibitor + DNA2 plasmid, and miR-3059-3p inhibitor + DNA2 plasmid groups were 3.4% ± 0.57%, 8.6% ± 0.60%, 30.2% ± 1.43%, 44.2% ± 3.9%, 15.4% ± 1.42%, 16.5% ± 2.05%, and 4.4% ± 1.18%, respectively. The miR-3059-3p mimic + DNA2 siRNA group had the highest percentage of apoptotic GBM8401 cells (Figure 6(b)).

### 3.4. miR-3059-3p Attenuated the HR Pathway to Reduce DNA Repair via Targeting DNA2

**Figure 7:**
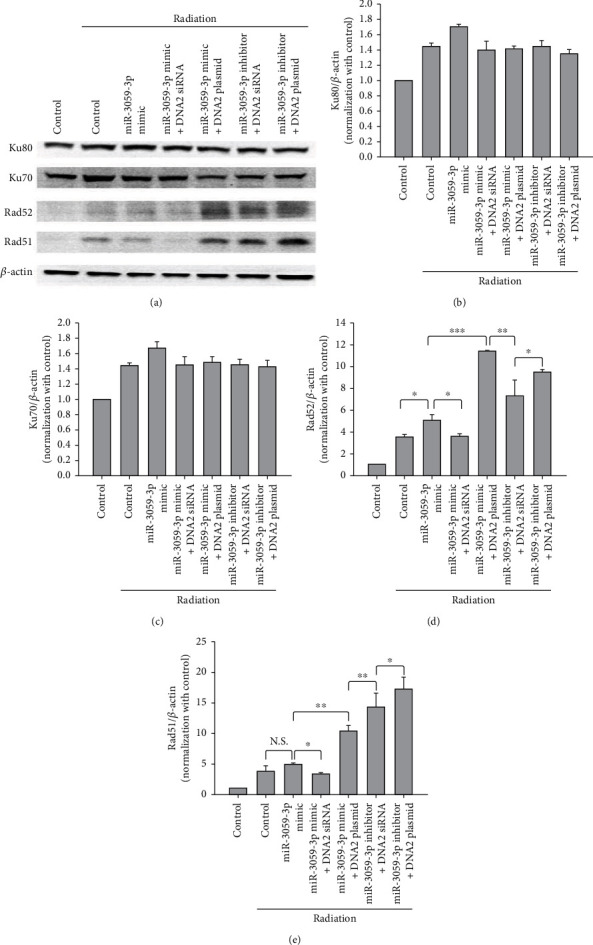
NHEJ and HR pathways were detected using immunoblotting. (a) Representative results of immunoblotting for Ku80, Ku70, Rad52, and Rad51. The Ku80 (b), Ku70 (c), Rad52 (d), and Rad51 (e) expression levels were normalized control group. ^∗^*P* < 0.05, ^∗∗^*P* < 0.01, and ^∗∗∗^*P* < 0.001.

Both HR and nonhomologous end-joining (NHEJ) are the main pathways in double-strand break (DSB) repair. RAD51 and RAD52 play key roles in HR pathway-mediated DNA repair. RAD51 was mediated on ssDNA in a form that is active for homologous pairing and strand invasion in humans. RAD51 also regulates dsDNA and prevents dissociation from ssDNA. RAD52 plays another crucial role in the repair of DNA DSBs at the active transcription sites during the G0/G1 phase of the cell cycle. Repair of these DSBs appears to use an RNA template-based recombination mechanism dependent on RAD52. In the NHEJ pathway, the KU70/80 heterodimer plays a vital role as it binds to DNA termini with high affinity, thereby protecting DNA ends from degradation, and recruits other NHEJ factors required for repair [25]. We used immunoblotting to determine which DNA repair pathway DNA2 could take. The results revealed that the protein expressions of Ku80 and Ku70 were similar in each group, but in the miR-3059-3p mimic and miR-3059-3p mimic + DNA2 siRNA groups, the protein expressions of both RAD52 and RAD51 were low. miR-3059-3p mimic + DNA2 plasmid, miR-3059-3p inhibitor + DNA2 siRNA, and miR-3059-3p inhibitor + DNA2 plasmid groups had high Rad52 and Rad51 protein expressions (Figure 7(a)). We found no significant difference in Ku80 and Ku70 expressions among the groups (Figures 7(b) and 7(c)). Moreover, in the miR-3059-3p mimic and miR-3059-3p mimic + DNA2 siRNA groups, the intensities of RAD52 and RAD51 were significantly lesser than those in other groups (Figures 7(d) and 7(e)) after radiation exposure. Our findings confirmed the association of miR-3059-3p with RAD52 and RAD51 and that miR-3059-3p increased radiosensitivity by targeting the DNA2 protein to affect the HR pathway in postradiation DNA repair.

## 4. Discussion

GBM in humans is still the most common primary malignant tumor of the central nervous system. Despite standard treatment including maximal surgical resection and RT with concomitant chemotherapy being well-established, the median progression-free and overall survival after the initial diagnosis is 6.2–7.5 and 14.6–20.5 months, respectively [26]. The main reason for these failures is the development of resistance to standard treatment regimens for GBM, including RR. Most studies over the years have elucidated the mechanisms of RR of GBM cells, and RR in these cells has been attributed to several mechanisms, including cell cycle, tumor microenvironment, hypoxia, apoptosis, cancer stem cells, microRNAs, and DNA damage and repair. In this study, RR cells exhibited downregulation of miR-3059-3p (Figure 1(a)) and upregulation of DNA2 (Figure 4(b)).

RT often results in DSB in cells. DNA damage response would induce RR in cancer cells. GBM cells develop RR via various DNA repair pathways, such as base excision repair, mismatch repair, nucleotide excision repair, homologous recombination repair, and NHEJ in glioma cells [27–29]. Inhibition of these pathways attenuated the RR cells and subsequent RT efficiency. Specific miRNAs can modulate proteins in the NHEJ pathway in gliomas. Blocking NHEJ-related proteins (KU70/KU80) was able to increase gene targeting efficiency [30].

The RAD51/RAD52 complex plays a key role in the HR pathway. Many studies have shown that inhibition of the HR pathway significantly enhances radiosensitivity in cancer cells. Chandler et al. showed that inhibition of Tat-associated T-cell-derived kinase-induced radiosensitivity through the HR pathway, not via the NHEJ pathway, in breast cancer [31]. Tang et al. showed that both ATM and EGFR inhibitors promote radiosensitivity through the HR pathway, not via the NHEJ pathway, in lung adenocarcinoma, cervical cancer, GBM, and colorectal carcinoma [32]. Our results revealed that inhibition of the HR pathway, not the NHEJ pathway, via miR-3059-3p enhances the therapeutic effects of radiation.

In this study, we found a relationship between targeting DNA2 protein and RAD51/RAD52 complex and that the DNA2 protein was attenuated via miRNA-3059-3p. DNA2 protein, which was first identified in yeast, plays an important role in DNA replication because of helicase and nuclease activities in the nucleus and mitochondria [33, 34]. DNA2 plays an important role in cell cycle, telomere maintenance, and DNA replication and repair [35]. Increased CHK1 expression has been shown to induce double-strand breaks (DSBs) through phosphorylation of DNA2 [36]. Gupta et al. showed that CHK1 inhibitor hypersensitizes osteosarcoma cells to radiation [37]. In our study, silencing DNA2 through miR-3059-3p targeting increased the percentage of apoptotic cells by inhibiting RAD51/RAD52 expression with radiation in GBM cells.

## 5. Conclusion

Currently, GBM remains a highly lethal cancer, despite several research efforts and clinical trials with agents designed to improve treatment outcomes. RR is among the reasons of treatment failure and tumor recurrence. Radiosensitizers have been considered and remain a viable option for improving the prognosis in patients with GBM. In our study, we focused on miR-3059-3p to target DNA2 and observed downregulated DNA2 expression. DNA2 plays an essential role in regulating the HR pathway and initiating DNA repair. Our data suggest that downregulation of DNA2 via miR-3059-3p could attenuate the HR pathway and decrease the possibility of DNA repair. Therefore, we believe that miR-3059-3p is an effective radiosensitizer candidate, which can inhibit GBM recurrence after RT.

## Data Availability

Data sharing is not applicable to this article as no datasets were generated or analyzed during the current study.
